# *Sophora japonica* Nanoemulsion: Ultrasound‐Assisted Extraction and Characterization

**DOI:** 10.1002/fsn3.70021

**Published:** 2025-01-31

**Authors:** Mohamed Ibrahim Younis, Ren Xiaofeng, Mohammad Ali Hesarinejad, Tarek Gamal Abedelmaksoud

**Affiliations:** ^1^ Food Science Department, Faculty of Agriculture Cairo University Giza Egypt; ^2^ School of Food and Biological Engineering Jiangsu University Zhenjiang China; ^3^ Research Institute of Food Science and Technology (RIFST) Mashhad Iran

**Keywords:** antioxidant activity, bioactive compounds, green extraction technologies, medicinal plant, nanoemulsions, oxidative stability

## Abstract

*Sophora japonica* , a member of the Fabaceae family, is recognized for its medicinal properties, particularly in traditional Asian medicine. This study aimed to investigate the efficiency of ultrasound‐assisted extraction techniques in obtaining bioactive compounds from *S. japonica* and to formulate these extracts into stable nanoemulsions with enhanced antioxidant activity. The extraction methods employed included ethanolic maceration followed by ultrasound‐assisted extraction, with the latter producing a total phenolic content of 65.57 mg gallic acid equivalent per milliliter, compared to 51.18 mg for the maceration method. Antioxidant activity was assessed using the DPPH scavenging assay, revealing that the ultrasound‐assisted extract exhibited a scavenging efficiency of 67%, whereas the ethanolic extract demonstrated a scavenging efficiency of 59%. The resultant nanoemulsions, formulated from the ultrasound‐assisted extracts, showed an average particle size of 252.92 nm and a zeta potential of −36.68 mV, indicating favorable stability. Visual inspections and peroxide value assessments during a 5‐day oxidative stability study indicated that the water‐based nanoemulsion experienced minimal changes, maintaining its stability, while the ethanolic nanoemulsion exhibited significant signs of separation and oxidation. These findings suggest that ultrasound‐assisted extraction not only enhances the recovery of bioactive compounds from *S. japonica* but also contributes to the formation of stable nanoemulsions, which hold potential applications in the food and pharmaceutical industries.

## Graphical abstract

Ultrasound‐assisted extraction extracts produce stable nanoemulsions with high quality. Ultrasound‐assisted extraction is more effective than ethanol extraction for extracting bioactive chemicals. Ethanol extract is more hydrophobic and oxidized than water extract.

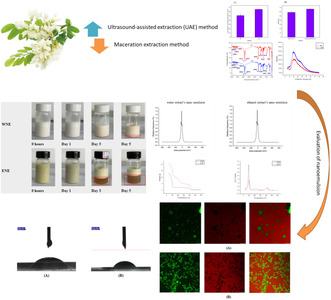

## 1 Introduction

*Sophora japonica* , also known as “zhong guohuai” or “huai shu,” is a species of shrub that is a member of the subfamily Faboideae (Yu, Yuan, and Chao 2021) of the pea family Fabaceae. Native to China, the provinces of Liaoning, Shaanxi, Shanxi, Shandong, Hebei, Henan, Jiangsu, Guangdong, and Guangxi are home to the majority of *S. japonica* . The Japanese pagoda tree, umbrella tree, and pagoda tree are some of the other names for it that are used in English. *S. japonica* is another herb used in traditional medicine to cool the blood and stop bleeding. Every component of this plant, including the flowers, buds, leaves, bark, and seeds, is used as medicine across Asia (Ungaro, Mehandru, and Allen 2017). Some of the primary components of *S. japonica* include flavonoids, isoflavonoids, triterpenes, phospholipids, amino acids, microelements, and polysaccharides (Wang, Wang, and Wang 2020; Yang, Sun, and Song 2021). Recent pharmacological studies have revealed that the active components and/or crude extracts of *S. japonica* exhibit a wide range of pharmacological actions, including cardiovascular effects as well as anti‐inflammatory, anti‐osteoporotic, antioxidant, antitumor, antibacterial, antiviral, hemostatic, and anti‐atherosclerotic effects (Gong, Fan, and Wang 2021; Karam, Petit, and Zimmer 2016; Bhatta, Janezic, and Ratti 2020).

Ultrasound, a recent green technology, boosts the speed and efficiency of several processes in the food processing industry. Additionally, it can be used with pressure (manosonication), temperature (thermosonication), or both to provide a synergistic effect that boosts its efficiency. Furthermore, ultrasound‐assisted extraction (UAE) is regarded as ecologically benign since it usually calls for lower solvent volumes and temperatures, which saves energy and minimizes solvent residues in the finished extracts (Bhargava, Mor, and Kumar 2021).

As such, fine particle size and increased stability of nanoemulsions have drawn interest in them as efficient delivery mechanisms for bioactive substances (Goswami, Rawat, and Pillai 2023). Nanoemulsions encapsulating phytochemicals not only increase their bioavailability and solubility but also shield them from oxidation and destruction (Mcclements and Rao 2011). Droplet sizes, stability, and other characteristics may all be greatly improved by ultrasonic emulsification (Mushtaq, Mohd Wani, and Malik 2023). Additionally, nanoemulsions offer versatility in applications, including pharmaceuticals, cosmetics, and functional foods.

While several studies have looked at how to extract bioactive components from *S. japonica* with ultrasonic assistance, not much has been done to see how to include these extracts into nanoemulsion formulations. Thus, the objective of this work is to find out how various properties of *S. japonica* nanoemulsions are affected by UAE. We shall assess in particular the antioxidant activity, stability, and physicochemical characteristics of the nanoemulsions made using extracts produced by UAE. Optimizing the formulation and investigating their possible uses in different sectors of *S. japonica* nanoemulsions requires an understanding of how extraction methods affect their characteristics.

## 2 Materials and Methods

### 2.1 Chemicals and Reagents

All chemicals and reagents used in this study were purchased from Sigma Chemical Co. Ltd. (St. Louis, MO, USA).

### 2.2 Preparation and Extraction

**FIGURE 1:**
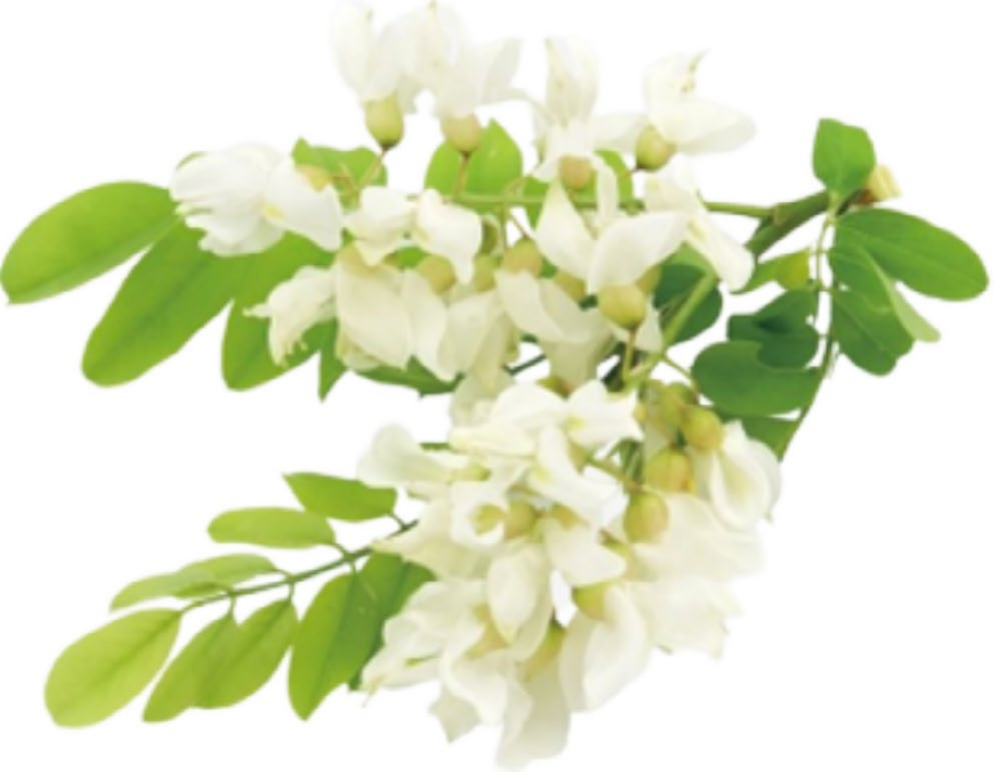
*Sophora japonica* flowers.

*S. japonica* flowers (Figure 1) were purchased from a local market in Zhenjiang, Jiangsu province, China, and stored in vacuum‐sealed bags at −20°C for 24 h. The flowers were subsequently freeze‐dried at −50°C and 0.1 mbar for 48 h, ground with a blender, and sieved through a 100‐mesh screen to obtain *S. japonica* flower powder. To prepare the extraction, 25 g of the powder were macerated with 250 mL of ethanol 70% which was reported by Li‐Na (2011). as the best ethanol concentration for extraction and left overnight at 1°C. The mixture was then filtered through a Whatman No. 1 filter to obtain the ethanol extract (Mao, Pan, and Que 2006).

Regarding UAE method, the used ultrasonic medium used were water or ethanol (70%), and based on (Gong, Li, and Li 2022), the best treatment duration was 15 min at a power level of 600 W, each treatment was repeated three times. The extracts were then stored in liquid form at 1°C.

### 2.3 Nanoemulsion Preparation

*Sophora japonica* flowers' extract was emulsified using a high‐speed homogenizer (IKA, T18 digital ultra turrax). A total of 50 mL of the extract was mixed with 100 mL of H_2_O containing 5% tween‐80 based on the weight of the extract (Ghazy, Fouad, and Saleh 2021). To ensure stability of the mixture, sonication was performed for 15 min at 600 W with ice cooling. The resulted nanoemulsion was then stirred for 24 h to eliminate ethanol (Gong, Li, and Li 2022). Finally, the nanoemulsion was frozen and prepared for the subsequent freeze‐drying process. In case of using 100% water, the same procedure was followed except without stirring for 24 h as it is ethanol‐free.

### 2.4 Evaluation of Maceration and UAE Extracts

#### 2.4.1 Total Phenolic Compounds

With few adjustments, the Folin–Ciocalteu technique was used to determine the total phenolic content (Minh 2021). Total of 5 mL of extract and 5 mL of 80% methanol were combined in a 15 mL centrifuge tube. The tubes were then centrifuged at 4000 rpm for 20 min at 4°C (Sigma 4‐16KS, Germany). For analysis, 100 μL of a standard solution or adequately diluted sample (10–100 g/mL) was combined with 3000 μL of deionized water, 100 μL of Folin–Ciocalteu reagent, and vortexed. Following a 10‐min incubation period at room temperature, 100 μL of a 20% sodium carbonate solution was immediately added, mixed, and then incubated for 2 h in the dark at room temperature. A microplate reader (Biotek Synergy 2 Microplate reader, USA) was then used to measure the mixture's absorbance at 765 nm. Total phenolic contents of the samples were expressed as mg of gallic acid per 100 mL using mg of gallic acid as the reference.

#### 2.4.2 DPPH Scavenging Assay

Antioxidant activity was assessed based on the ability to scavenge free radicals while interacting with a stable DPPH free radical according to Česonienė, Liaudanskas, and Žvikas (2022).

Antioxidant activity was calculated as % of radical scavenging percentage using Equation (1):

(1) $$\%\text{radical scavenging percentage}\;=\;\frac{A0\;-\;A1}{A0}\;\times 100$$

where A0 = the absorbance of the control reaction (containing all reagents except the test compounds [*t* = 0 min]). A1 = absorption of test extract solution (*t* = 30 min).

#### 2.4.3 Colorimetric Analysis

The color of the extracts was evaluated using a colorimeter (Sucolor, China). The total chromaticity difference (*E*) was calculated using an equation utilizing the L* (lightness), a* (red‐green), and b* (yellow‐blue) values:

(2) $$\Delta E\;=\;{\left(\Delta L^{*2}\;+\;\Delta a^{*2}\;+\;\Delta b^{*2}\right)}^{1/2}$$

By deducting the standard color parameters from the corresponding L*, a*, and b* values, the values of Δ*L*, Δ*a*, and Δ*b* were determined. The total chromaticity difference (Δ*E*) was then calculated as the square root of the sum of the squared differences in L*, a*, and b*. The calibrated whiteboard values were L* = 89.03; a* = 0.35; b* = 3.42.

#### 2.4.4 Attenuated Total Reflectance–Fourier Transform Infrared

The Fourier transform infrared (FTIR) spectra of dried samples were obtained using an FTIR spectrometer (Nicolet iS50, Thermo Electron, USA) that was equipped with a diamond crystal attenuated total reflection (ATR) accessory. The spectra were collected within the range of 4000–500 cm^−1^ with a resolution of 4 cm^−1^ and the number of scans was set to 16 (Kang, Liang, and Chen 2023).

#### 2.4.5 X‐Ray Diffraction

The crystal structures of different dry samples were analyzed using a Bruker D8 Advance x‐ray diffractometer (XRD) from Germany. The samples were placed in the center and flattened before testing. The scanning range for 2θ was set to 5°–40°, with a rate of 5° per minute. The diffraction patterns were collected and analyzed for crystal structure determination.

### 2.5 Evaluation of Nanoemulsion

#### 2.5.1 Particle Size and Zeta Potential

In order to determine the particle size and zeta potential of *S. japonica* UAE nanoemulsion, a laser particle size analyzer (Litesizer 500, Anton Paar, Austria) was used. Particle size of the sample was measured in a throwaway sample cell at a temperature of 25°C, after diluting the sample solution with distilled water while solvent's refractive index was 1.33. Additionally, the zeta potential was detected using an Omega II cell. These measurements provided indications about nanoemulsion stability and potential applications.

#### 2.5.2 Thermogravimetric Analysis

A SDT Q600 (V20.9 Build 20) was used to perform the thermogravimetric analysis (TGA) of *S. japonica* UAE 70% ethanol extract's nanoemulsion (ENE). Approximately 20 mg sample was measured and tightly packed in aluminum containers. The samples were elevated from 30°C to 600°C at a rate of 10°C/min while being flushed with nitrogen at a rate of 30 mL/min. The reference was an empty aluminum container (Xu, Han, and Liu 2023). The sample was compared with UAE 100% water extract's nanoemulsion (WNE) to evaluate the effect of ethanol on stability.

#### 2.5.3 Visual Inspection and Stability

To evaluate the extent of lipid oxidation in the emulsions, fresh samples were placed in airtight glass jars and stored in an oven at 45°C for a period of 5 days to allow for self‐oxidation. The visual inspection was performed to evaluate the visual change in relation to the stability of the emulsions.

The measurement of peroxide value (POV) was used to assess lipid hydrogen peroxide and secondary oxidation products in the oils. This was accomplished by analyzing the resulting samples for POV levels. The experimental setup was designed to ensure accurate measurement of lipid oxidation in the emulsions (Walker, Gumus, and Decker 2017).

#### 2.5.4 Confocal Laser Scanning Microscopy

The morphology of the emulsions was characterized using a confocal laser scanning microscope (CLSM) (Leica TCS SP5, Germany). CLSM was used to examine the emulsion micromorphology. Specifically, 10 μL of the emulsion was deposited on a microscope slide, covered with a coverslip, and observed using a 100× oil immersion objective lens at a temperature of 25°C. All images were acquired at a 200 Hz scanning frequency and 1024 × 1024 scanning density (Ren, Zhou, and Qayum 2023).

#### 2.5.5 Contact Angle

Contact angles were measured using a contact angle analyzer (DCAT21, Data Physics, Germany) through the static drop method. Thin layers of WNE and ENE suspensions were prepared and air‐dried for 4 h. The samples were then placed in an environment with 25°C temperature and 65% relative humidity for 24 h to achieve equilibrium. Powder was prepared using the tableting method. The contact angles were determined from captured droplet images by applying the Young–Laplace equation (Dai, Chen, and Zhang 2021).

### 2.6 Statistical Analysis

The study utilized SPSS software for statistical analysis with experiments conducted in triplicate. Duncan's multiple comparison tests (*p* < 0.05) were employed to compare means and determine statistical significance.

## 3 Results and Discussion

### 3.1 Evaluation of Maceration and UAE Extracts

#### 3.1.1 Total Phenolic Compounds and DPPH Scavenging Assay

**FIGURE 2:**
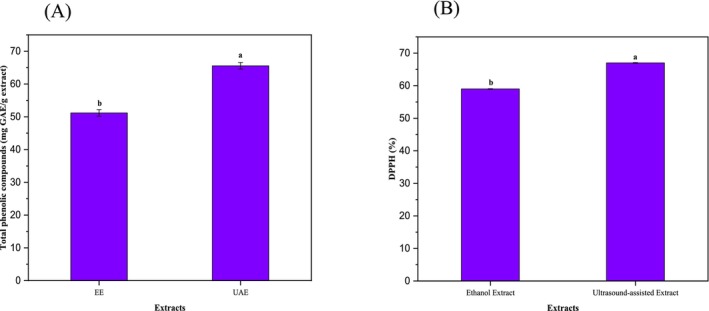
(A) Total phenolic compounds after using different extraction methods. (B) DPPH percentages of different extracts. a and b represent the significance letters between the treatments.

Total phenolic content was determined for the two extracts (ethanolic maceration extraction [EE] and UAE) as shown in Figure 2A. The results for EE and UAE were 51.18 and 65.57 mg GAE/mL extract, respectively. The higher value for UAE extract compared to EE extract could be caused by the fact that UAE was more efficient method for extracting phenolic compounds from *S. japonica* . The high phenolic content observed for the UAE extract suggests that it could be a potential source of natural antioxidants that can be used in the food and pharmaceutical industries and that UAE is a more efficient method for the extraction of phenolic compounds as mentioned before in previous studies (Guandalini, Rodrigues, and Marczak 2019; Lobo, Nascimento, and Domingues 2017). EE sample showed a DPPH scavenging activity of 59%, while UAE sample showed a scavenging activity of 67% (Figure 2B). This suggests that UAE sample has a higher antioxidant activity compared to EE sample. Although the two samples were extracted from the same plant species, the higher scavenging activity of UAE sample is attributed to a higher concentration of antioxidant compounds. These DPPH results are consistent with TPC results and previous studies (Liu, Liu, and Xia 2021), which have reported the presence of various bioactive compounds with antioxidant properties are higher in UAE sample. The findings suggest that *S. japonica* flowers have the potential to be used as a natural source of antioxidants.

#### 3.1.2 Colorimetric Analysis

The colorimetric data (Table 1) shown that there were considerable color changes between the two samples and the reference standard.

**TABLE 1:** Colorimetric characteristics values.

Extract	ΔL*	Δa*	Δb*	Δ*E*
Maceration (EE)	−59.48	14.79	2.87	61.36
UAE	−63.47	10.98	−0.57	64.41

As the ΔL* value of the UAE sample (−63.47) was lower than the ΔL* value of the EE sample (−59.48), indicating that the UAE sample was darker than the EE sample. For Δa* value, the EE sample (14.79) was higher than UAE sample (10.98), indicating more redness. While for Δb* value, the EE sample (2.87) was higher than UAE sample (−0.57), indicating more yellowness.

Total color difference (ΔE) for UAE sample (64.41) was slightly higher than EE sample (61.36), indicating a slightly greater overall color difference from the reference standard.

Based on these results, it was noticed that the UAE sample was darker and had less redness and more blueness than the EE sample and that these differences may be due to using ultrasound treatment results in more extracted compounds which was similar to results from previous studies (Da Rocha and Noreña 2020).

#### 3.1.3 ATR–FTIR of Extracts

**FIGURE 3:**
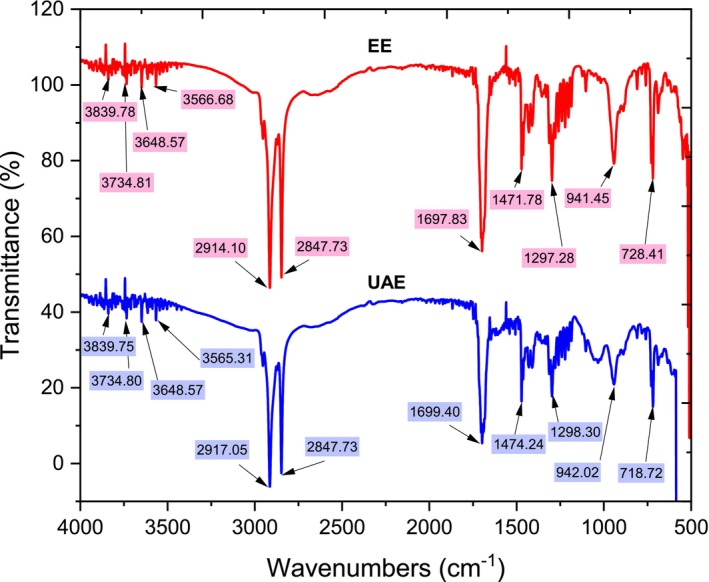
ATR–FTIR spectrum of EE and UAE samples.

A comparison of the FTIR spectra obtained for *S. japonica* EE and UAE revealed some noticeable differences. As shown in Figure 3, some peaks in the UAE were either absent or shifted when compared to the EE. The most significant differences were observed in the peaks at 2917.05 and 1699.40 cm^−1^. For 2917.05 cm^−1^, was attributed to the stretching vibration of the hydroxyl (—OH) group in alcohols and phenols. While for 1699.40 cm^−1^, the difference was due to the C=O stretching vibration of esters and carboxylic acids. It was expected that the intensities vary slightly depending on the treatment but it does not appear to relate to chemical shifts or to peaks appearance or absence (Liu, Renard, and Bureau 2021).

#### 3.1.4 XRD of Extracts

**FIGURE 4:**
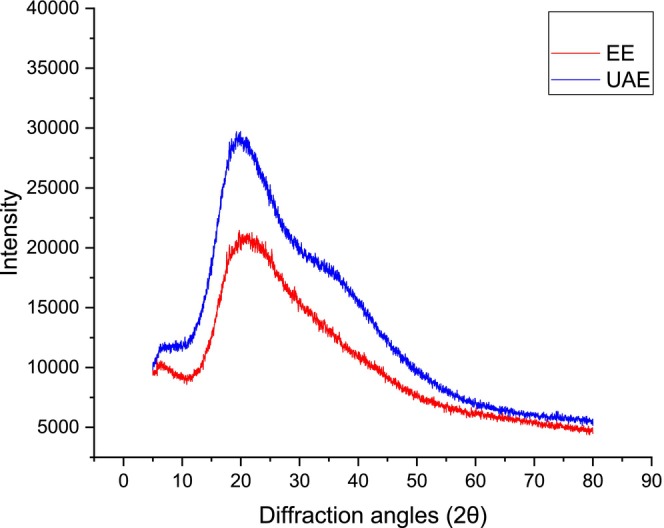
X‐ray diffraction spectrum of extracts after using different extraction methods.

The XRD diffraction patterns for both UAE and EE extracts were recorded in the range of 5–50. As shown in Figure 4, the XRD pattern for UAE showed a sharp peak at 19.23° in addition to peaks at 8.87° and 31.82° with crystallinity percentage of 31.23%. On the other hand, the XRD pattern for EE reflected two distinct peaks at 2θ value 6.35° and 19.93°, with the latter peak appearing much sharper at 19.75° and crystallinity percentage of 37.64%. These results were consistent with the findings reported in previous studies (Hasani, Ojagh, and Ghorbani 2018).

The changes in the XRD pattern for UAE following ultrasonic treatment indicated an improvement in the molecular arrangement and enhanced intermolecular forces, resulting in a more compatible macromolecular structure which was proven in previous studies (Qu, Guo, and Zhang 2022; Liu, Wang, and Kang 2018; Liang, Chen, and Ren 2021; Asgari, Labbafi, and Khodaiyan 2020).

### 3.2 Nanoemulsion Evaluation

#### 3.2.1 Total Phenolic Compounds and Antioxidant Activity

The nanoemulsions' total phenolic contents are shown in Table 2. The TPC values in ENE were slightly lower at 57 mg GAE/g, but much higher in WNE at 61 mg GAE/g. The absorbance drop at 517 nm was used to determine how much reduction DPPH radicals may undergo. Because the antioxidant may donate and scavenge hydrogen, DPPH radical absorption decreases. DPPH assay showed that WNE had the highest scavenging with 67% followed by ENE with 61% as shown in Table 2. β‐carotene/linoleic acid assay was used to screen the nanoemulsions for possible antioxidant activity. The results in Table 2 unequivocally reveal that WNE performed better than ENE in the β‐carotene/linoleic acid test with a value of 95.36%.

**TABLE 2:** Total phenolic content, DPPH, and β‐carotene/linoleic acid of nanoemulsions.

Samples	TPC (mg GAE/g)	Antioxidant activity	
		DPPH (%)	β‐carotene/linoleic acid (inhibition ratio %)
WNE	61 ± 0.12a	67 ± 0.14a	95.36 ± 0.27a
ENE	57 ± 0.23b	61 ± 0.15b	90.12 ± 0.22b

a and b represent the significance letters among the treatments.

#### 3.2.2 Particle Size and Zeta Potential

Litesizer 500 was used to determine the particle size of UAE nanoemulsion. The test results revealed that the average hydrodynamic radius of the particles was 252.92 nm at a temperature of 25°C (Table 3).

**TABLE 3:** Hydrodynamic radius of nanoemulsions.

Sample	Temperature (°C)	No. runs	Hydrodynamic radius (nm)			
			Rep. 1	Rep. 2	Rep. 3	Average
WNE	25	30	274.59	238.83	245.35	252.92
ENE	25	30	281.43	260.07	253.29	264.93

This finding indicates that UAE was composed of particles with a significant size distribution, which could affect their physicochemical characteristics, such as solubility, stability, and reactivity (Said, Sundar, and Tiwari 2022).

**FIGURE 5:**
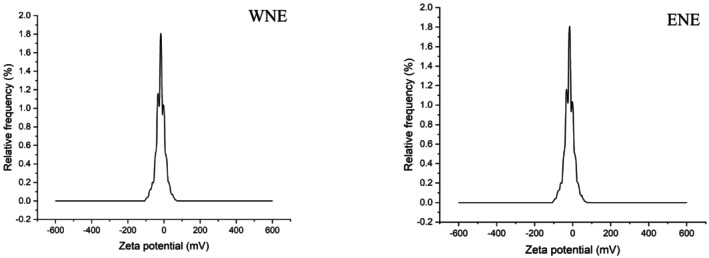
Zeta potential of WNE and ENE.

On the other hand, zeta potential values for WNE and ENE were determined to be −35.45 and −36.68 mV (Figure 5), respectively, which shows that the system is less prone to aggregation and that the particles are evenly distributed, which may improve efficiency and usefulness (Sezer, Atieh, and Koç 2019).

Additionally, various research studies have provided evidence indicating that the stability of emulsion formation is positively correlated with lower particle size values (Wang, Ding, and Zhao 2021), as well as higher zeta potential of nanoparticles (Lu and Tian 2021).

#### 3.2.3 Thermogravimetric Analysis

**FIGURE 6:**
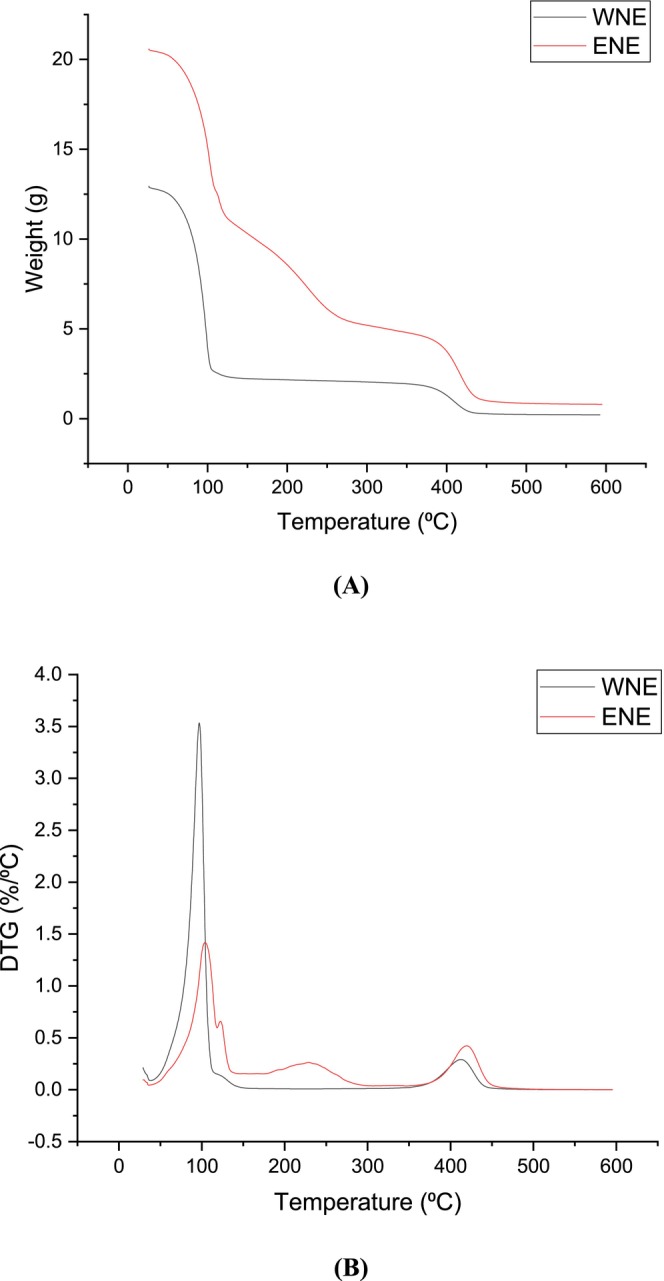
(A) TGA spectrum of WNE and ENE. (B) DTG spectrum of WNE and ENE.

TGA/DTG thermal curves of 70% ethanol emulsion (ENE) and 100% water emulsion (WNE) from 30°C to 600°C were generated to visualize weight loss and maximum decomposition temperature (Figure 6A). In the first stage of thermal degradation occurred at 30°C–340°C for ENE and 30°C–265°C for WNE due to the evaporation of liquids from the emulsion. The TGA curve demonstrated that the weight loss of the WNE was greater than that of ENE (Table 4). This suggests that higher water concentration results in greater weight loss at the first degradation stage. The DTG curves revealed that the water loss temperatures of ENE and WNE were 108.5°C and 102.66°C, respectively.

**TABLE 4:** Thermogravimetric characteristics of nanoemulsions.

Sample	Weight loss% during each degradation stage		Total weight loss%
	First stage	Second stage	
WNE	83.06	14.3	97.36
ENE	75.91	19.55	95.46

The second thermal degradation stage of both ENE and WNE was observed to occur in the temperature range of 340°C–505°C and 265°C–472°C, respectively. This degradation was mainly attributed to the breakdown of covalent bonds, electrostatic interactions, and hydrogen bonds (Dutta, Nashre‐Ul‐Islam, and Saha 2019). Notably, the thermal decomposition temperature (Td) of ENE was found to be 420.13°C higher than that of WNE, as evidenced in Figure 6B. These results suggest that ultrasound treatment facilitated the formation of more ordered and stable structures, thereby improving the thermal stability of the emulsions (Asadi, Pourfattah, and Miklós Szilágyi 2019). Additionally, a minor weight loss was observed during the second stage of thermal degradation, indicating enhanced inter‐molecular interactions (Maher, Kabbashi, and Mirghani 2021).

#### 3.2.4 Visual Inspection and Stability

**FIGURE 7:**
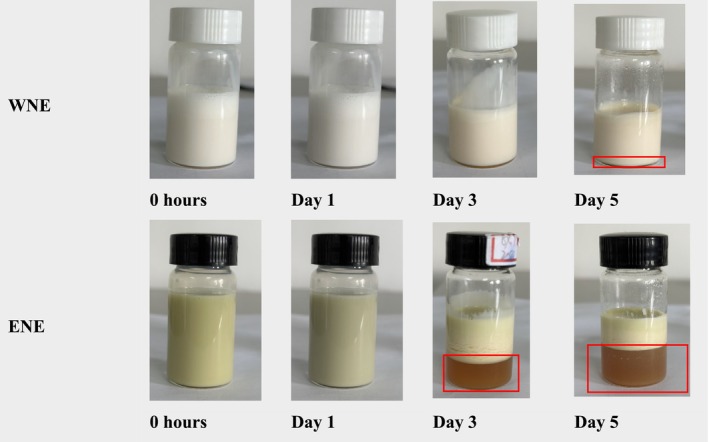
Visual inspection of water extract nanoemulsion (WNE) and ethanol extract nanoemulsion (ENE) through 5 days of storage at 45°C.

**FIGURE 8:**
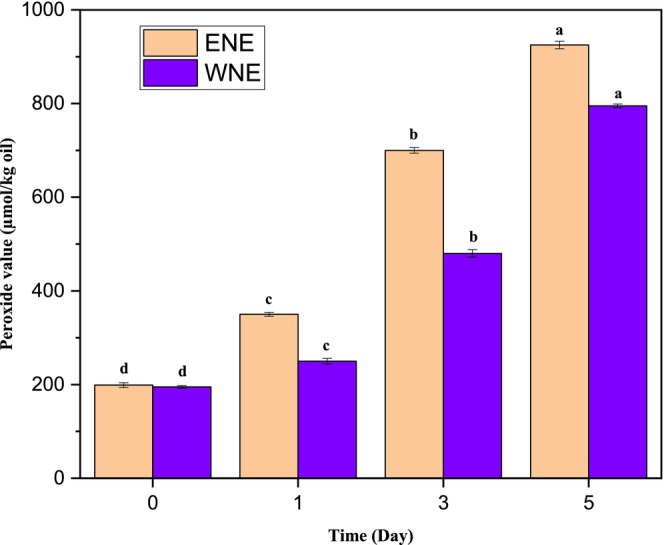
Peroxide value of both water extract nanoemulsion (WNE) and ethanol extract nanoemulsion (ENE) through 5 days of storage at 45°C. a, b, c and d represent the significance letters among the treatments.

The effect of oxidative stability on oil in nanoemulsion was assessed through visual inspection (Figure 7) and measurement of POV (Figure 8). The physical changes observed in the WNE over a period of 5 days indicated that the emulsion was stable, with only a slight color change after the first day, suggesting the onset of oxidation followed by a noticeable color change indicating an increase in the oxidation rate. In contrast, the ENE showed a significant color change after the first day, followed by a clear separation at the bottom of the vial after the third day, and an increased separation volume after the fifth day, which indicated a rapid increase in oxidation rate. The POV values also increased gradually with time, indicating that the essential oil in both emulsions was undergoing oxidation. After 5 days, the POV contents in WNE only increased from 480.39 to 693.06 μmol/kg oil, while the POV contents in ENE increased from 633.75 to 925.55 μmol/kg oil deducting that the rate of increase in POV for WNE was significantly slower than that of ENE. These results suggest that WNE was more stable and effectively retarded the oxidation of oils in the emulsion (Song, Zheng, and Ma 2020).

#### 3.2.5 Confocal Laser Scanning Microscopy

**FIGURE 9:**
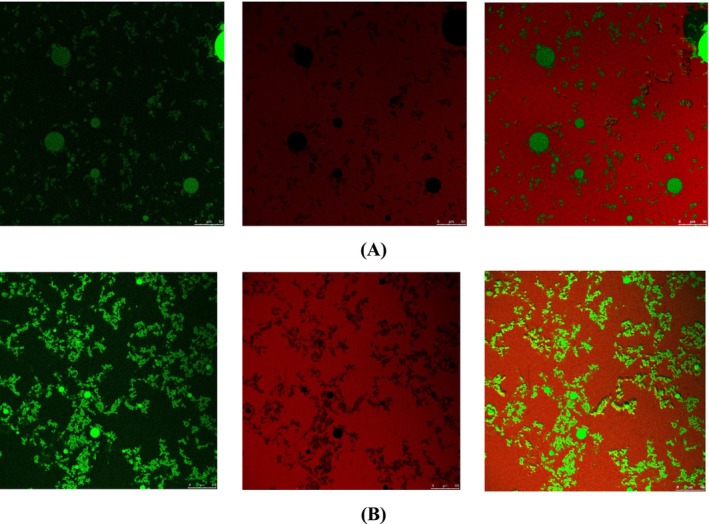
(A) CLSM image of WNE. (B) CLSM image of ENE.

*Sophora japonica* WNE and 70% ENEs were prepared and characterized using CLSM imaging. The CLSM results of *S. japonica* WNE showed small, dispersed green globules throughout the sample, indicating that the water‐soluble components of the plant extract have formed a stable nanoemulsion in water (Figure 9A). This is in line with previous studies that have reported the presence of phenolic compounds and flavonoids in the water extract of *S. japonica* (Jiang, Song, and Zhao 2022).

On the other hand, the CLSM imaging of the 70% ENE showed larger numbers, aggregated green globules that appeared to be clustered together (Figure 9B). This could be attributed to the higher concentration of nonpolar compounds, such as alkaloids or terpenoids which are soluble the ethanol (Falsafi, Rostamabadi, and Assadpour 2020).

#### 3.2.6 Contact Angle

**FIGURE 10:**
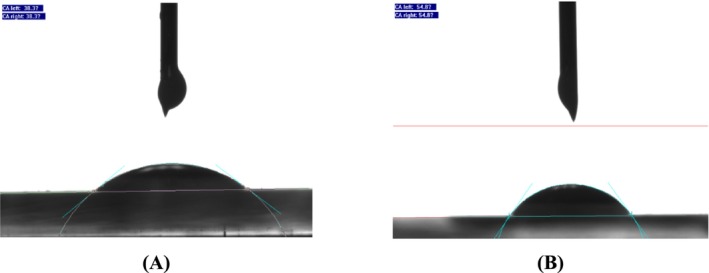
(A) Contact angle of WNE. (B) Contact angle of ENE.

The results obtained from the contact angle test (Figure 10) showed that WNE had a contact angle of 38.3° on both the left and right sides, indicating a relatively hydrophilic (i.e., water‐loving) surface. This result suggests that the surface may contain polar or charged groups or may have a rough texture that promotes wetting. In contrast, ENE had a contact angle of 54.8° on both the left and right sides, indicating a relatively hydrophobic (i.e., water‐repelling) surface. This result suggests that the surface may contain non‐polar or hydrophobic groups or may have a smooth texture that inhibits wetting which is similar to previous studies (Pirozzi, Del Grosso, and Ferrari 2020).

## 4 Conclusion

In recent years, there has been a growing interest in enhancing the extraction and application of bioactive compounds from natural sources, particularly for their potential health benefits. This study highlights the importance of incorporating advanced extraction methods, such as UAE, to improve the yield and stability of compounds derived from *S. japonica* . The results indicate that UAE not only outperforms traditional methods in terms of total phenolic content but also enhances the antioxidant capacity of the extracts. By producing nanoemulsions with favorable characteristics, this methodology opens new avenues for utilizing *S. japonica* in various sectors. The observed stability of the generated nanoemulsions suggests their viability for incorporation into dietary supplements and functional foods, providing consumers with enhanced health benefits. Furthermore, the findings of this research pave the way for further investigations into other potential uses of UAE. Future studies could explore different solvents, extraction times, and temperatures to optimize the yield of other beneficial compounds, as well as investigate the synergistic effects of combining multiple extracts. Ultimately, this research not only demonstrates the effectiveness of modern extraction techniques but also emphasizes the need for continued exploration into the application of plant‐derived active ingredients as functional additives in diverse industries.

## Author Contributions

Mohamed Ibrahim Younis, Ren Xiaofeng, Mohammad Ali Hesarinejad, and Tarek Gamal Abedelmaksoud contributed to the design and implementation of the research, to the analysis of the results, and to the writing of the manuscript.

## Conflicts of Interest

The authors declare no conflicts of interest.

## Data Availability

All data generated or analysed during this study are included in this published article.
